# Nutri-Score and NutrInform Battery: Effects on Performance and Preference in Italian Consumers

**DOI:** 10.3390/nu14173511

**Published:** 2022-08-26

**Authors:** Morgane Fialon, Mauro Serafini, Pilar Galan, Emmanuelle Kesse-Guyot, Mathilde Touvier, Mélanie Deschasaux-Tanguy, Barthélémy Sarda, Serge Hercberg, Lydiane Nabec, Chantal Julia

**Affiliations:** 1 Nutritional Epidemiology Research Team (EREN), Sorbonne Paris Cité Epidemiology and Statistics Research Center (CRESS), Inserm U1153, Inrae U1125, Cnam, Université Sorbonne Paris Nord University, 93017 Bobigny, France; 2 Faculty of Biosciences and Technologies for Agriculture, Food and Environment, University of Teramo, 64100 Teramo, Italy; 3 Public Health Department, Avicenne Hospital, Assistance Publique des Hôpitaux de Paris (AP-HP), 93017 Bobigny, France; 4 Centre de Recherche Réseaux, Innovation, Territoire et Mondialisation (RITM), Université Paris-Saclay, 91190 Gif-sur-Yvette, France

**Keywords:** front-of-pack, Nutri-Score, NutrInform Battery, Italy

## Abstract

In May 2020, the European Commission announced a proposal for a mandatory front-of-pack label (FoPL) for all European Union (EU) countries. Indeed, FoPLs have been recognized by several public institutions as a cost-effective measure to guide consumers toward nutritionally favorable food products. The aim of this study was to compare the performance and consumer preference of two FoPLs currently proposed or implemented in EU countries, the interpretive format Nutri-Score and the non-interpretive format NutrInform Battery, among Italian consumers. The experimental study was conducted in 2021 on a representative sample of 1064 Italian adults (mean age = 46.5 ± 14.1 years; 48% men). Participants were randomized to either Nutri-Score or NutrInform and had to fill out an online questionnaire testing their objective understanding of the FoPL on three food categories (breakfast products, breakfast cereals and added fats) as well as purchase intention, subjective understanding and perception. Multivariable logistic regressions and *t*-tests were used to analyze the answers. In terms of the capacity of participants to identify the most nutritionally favorable products, Nutri-Score outperformed NutrInform in all food categories, with the highest odds ratio being observed for added fats (OR = 21.7 [15.3–31.1], *p* < 0.0001). Overall, with Nutri-Score, Italian participants were more likely to intend to purchase nutritionally favorable products than with NutrInform (OR = 5.29 [4.02–6.97], *p* < 0.0001). Focusing on olive oil, participants of the Nutri-Score group had higher purchase intention of olive oil compared to those in the NutrInform group (OR = 1.92 [1.42–2.60], *p* < 0.0001) after manipulating the label. The interpretive format Nutri-Score appears to be a more efficient tool than NutrInform for orienting Italian consumers towards more nutritionally favorable food choices.

## 1. Introduction

Dietary intakes of added/free sugars, saturated fatty acids (SFA) and sodium are considered excessive, and intakes of dietary fiber and potassium insufficient, compared with current recommendations in a majority of European countries including Italy [1,2]. These inadequate intakes are known to cause potential adverse health effects [3] such as obesity. In Italy, where adherence to the Mediterranean diet is declining, especially among younger generations [4,5,6], nearly one-third of children are overweight or obese [7]. Front-of-Pack Labels (FoPLs) have been identified by the Organization for Economic Co-operation and Developments (OECD) [8] and the World Health Organization (WHO) [9] as an efficient policy tool to tackle noncommunicable diseases and to cope with deteriorating consumption habits. Since the 1980s, several formats of FoPLs have been proposed by European stakeholders such as the Green Keyhole in Nordic countries [10], the Multiple Traffic Lights in the United Kingdom [11], or the Reference Intakes implemented by food and drink manufacturers in 2006 [12]. More recently, the Nutri-Score was adopted in France in 2017 and then in several other countries. The Nutri-Score, designed by academic researchers and the French Public Health Agency [13], is a summary, graded, color-coded front-of-pack nutrition label providing an overall appreciation of the nutritional value of pre-packed products (with five categories from dark green/A to dark orange/E). The color/letter is attributed on the basis of an algorithm considering, for 100 g or 100 mL of product, the content of nutrients to be limited (energy, saturated fatty acids, sugars, salt) and of nutrients and foods to be favored (fiber, proteins, and percentage of fruits, vegetables, legumes, nuts, rapeseed, walnut and olive oils). Several scientific studies have validated the nutritional algorithm underlying the Nutri-Score, as well as its ability to guide consumers towards more nutritionally healthy food choices [14]. However, in Italy, the main political and economic stakeholders involved in the food and agriculture sector have shown a strong opposition to Nutri-Score, positioning it as a threat to traditional Italian food products and the Mediterranean diet [15]. Following this controversy, the FoPL NutrInform Battery, with a format similar to the existing Reference Intakes, was developed by four Italian ministries and officially adopted in Italy in 2020. The NutrInform Battery [16] is a non-interpretive FoPL displaying the content of energy, fats, saturated fats, sugars and salt per serving as well as the corresponding contribution to the daily dietary requirements of an average adult represented in the form of battery symbols. Nutri-Score and NutrInform represent two different strategies in terms of front-of-pack labeling. The first is interpretive and provides an assessment of the relative nutritional value of a food product based on the information available at the back-of-pack, the second is non-interpretive and reproduces part of the information available from the back-of-pack labeling, without additional interpretation. The WHO European Regional Office stated that “interpretive FoPL [17] is seen as a cost-effective measure to help consumers understand the nutritional quality of foods, and orient them towards healthier food choices at the point of purchase” [17]. From an economic perspective, an OECD report concluded that “population-wide interventions such as food labelling, menu labelling and mass media campaigns will produce the largest health gains and largest savings in health expenditure” [18]. In line with these international recommendations, the European Commission stated it would propose a harmonized mandatory front-of-pack nutrition label for the EU in 2022 as part of the Farm to Fork strategy [19]. To date, no studies have directly compared the performance of Nutri-Score and NutrInform in terms of consumers’ objective understanding and purchase intention. Available studies on these two FoPLs [20,21,22] have remained centered on subjective understanding and perception which are by definition not objective measurements. Thus, the aim of this study was to compare the performance of Nutri-Score and NutrInform among a sample of 1064 Italian consumers through objective understanding and intention to purchase nutritionally favorable products, as well as preference through subjective understanding and perception, following an experimental design.

## 2. Materials and Methods

### 2.1. Participants

Between 17 November 2021 and 3 January 2022, 1064 Italian participants were recruited by the ISO-accredited international web panel provider PureProfile to perform an online questionnaire on Nutri-Score and NutrInform. This specific sample allowed us to follow quotas on age, sex and education level of the general Italian population [23]. The protocol of the present study was approved by the Institutional Review Board of the French Institute for Health and Medical Research (n°22-876).

### 2.2. Design and Stimuli

To test Front-of-Pack Labels (FoPLs), three food categories (breakfast products, breakfast cereals and added fats) were selected for their high variability in nutritional quality within the category and/or because they have been the subject of controversy in the current FoPLs debate (one of the main critics is the classification of olive oil by Nutri-Score [24]). Moreover, breakfast is an eating occasion including a more limited choice of products compared to other meals and is therefore suitable to model consumer choice strategies. Images of real-life products that could be bought in Italian supermarkets were used (Figure S1). FoPLs were positioned below the food product image, with a zoom function available. The first food category, breakfast products, included eight products commonly consumed for breakfast such as biscuits, breakfast cereals or crispbread. The second food category focused on breakfast cereals only with seven products such as chocolate-flavored cereals or oat flakes (Table S1). The last food category comprised eight added fats including various vegetable oils (e.g., olive oil, sunflower oil) and butter. All products’ images of the same food category were displayed on the same screen page and were approximately the same size. Back-of-pack information such as ingredients or nutritional values was not available in order to avoid information overload and to mimic in-store presentation of products, in which back-of-pack information is not visible.

### 2.3. Procedure

Completion of the online questionnaire took about 20 min. Eligible participants were asked to provide information on their sex, age, educational level, household composition, self-estimated level of nutrition knowledge and diet quality. FoPL context was also captured by asking respondents if they had heard about Nutri-Score or NutrInform before the survey and if what they had heard was positive, neutral or negative. Following this general section, participants were randomly assigned to either Nutri-Score or NutrInform groups. As a first step, respondents had to read an information note detailing the characteristics of the FoPL they were assigned to (Table S2) and then had to assess seven statements testing their understanding of the information note (four-point Likert scale: “strongly disagree”; “somewhat disagree”; “somewhat agree”; “strongly agree” with an “I don’t know” option available).

Participants were then presented successively with each food category: breakfast products (8 products), breakfast cereals (7 products) and added fats (8 products). For each food category, participants had to respond, through a four-point Likert scale (same as mentioned previously), if they felt that the FoPL was helping them to differentiate the nutritional qualities of food products, as a proxy for **subjective understanding**. As a proxy for **objective understanding**, participants were then tasked to select the three products (one product for added fats) that they considered were the most nutritionally favorable, placing the best one first, for the three food categories mentioned above (breakfast products, breakfast cereals and added fats). Regarding **purchase intention**, participants were asked which product they would purchase most frequently. **Perception** of the FoPL was assessed through 13 statements grouped into the following four dimensions afterwards, on which participants had to give their opinion through a nine-point Likert scale (1: “strongly disagree”; 5: “neither agree nor disagree”; 9: “strongly agree”):

a. *Ease of use*. Measured through: “this label helps me to make better food choices”, “this label is a source of confusion for me in my food choices”, “this label is easy to interpret”, and “this label is easy to understand”.
b. *Capacity to inform*. Measured through: “this label provides me with the information I need to make my food choices”, “this label does not provide me with any information about the nutritional quality of food products”, “this label is useful for informing me about the nutritional quality of food products” and “this label is effective in informing me about the nutritional quality of food products”.
c. *Trust*. Measured through: “this label is credible and inspires confidence”, “I feel I can count on this label to inform me about the nutritional quality of food products” and “if I don’t know the food product, I can always count on this label to inform me about its nutritional quality”.
d. *Liking*. Measured through: “I like this label” and “I do not want this label to be placed on foods”.

Finally, at the end of the questionnaire, participants could see both Nutri-Score and NutrInform on three types of cookies and had to select the FoPL they thought the easiest (direct preference 1) and the fastest (direct preference 2) to evaluate the nutritional quality of the products presented. Of note, this last part of the questionnaire was the only moment participants were exposed to both labels and had to provide a **direct preference** towards one of them.

### 2.4. Statistical Analysis

Sociodemographic and FoPL contextual data were summarized per randomization group. Understanding of the information note was analyzed on a grade out of seven and compared through a Welch’s *t*-test (unequal variances). **Subjective understanding** per food category was assessed by converting the four-point Likert scale into a score ranging from −2 (strongly disagree) to +2 (strongly agree) with a 0-score allocated to “I don’t know” answers. A mean score per food category and per FoPL was calculated and compared through Welch’s *t*-test. 

For general **perception** of the FoPL, answers for the three statements: “this label is a source of confusion for me in my food choices”; “this label does not provide me with any information about the nutritional quality of food products” and “I do not want this label to be placed on foods” were reversed in order to give all 13 statements the same orientation. In addition, participants who responded “neither agree nor disagree” to all statements of a perception group were excluded from the statistical analysis. A Principal Component Analysis (PCA) was performed for each group mentioned above (*Ease of use, Capacity to inform*, *Trust* and *Liking*). The first PCA dimension was retained as it accounted for an adequate level of variance (69%, 69%, 87% and 70% for each perception group, respectively) and comparisons of mean coordinates between the two randomization groups were conducted using Standard/Welch’s *t*-tests (according to variance).

Regarding **objective understanding**, responses on the three most nutritionally favorable products were analyzed through two variables: the *one-product task,* which was considered correct if the participant put the expected product in the first position (1 point); the *three-product task,* scored from 3 points when the participant chose the three expected products (regardless of the order) to 0 point if the participant selected none among correct ones. To ensure equitable assessment of the labels, expected products were defined according to the FoPLs specificities. In the case of Nutri-Score, the assessment was based on the Nutri-Score’s grades. In the case of NutrInform, we classified products according to the sum of percentages indicated in the batteries (except total fat). Products with the lowest sums were considered the most nutritionally favorable as daily intakes of energy, saturated fats, sugars and salt are maximum values that should not be exceeded. Depending on the food category, expected responses could be different for each randomization group (Table S1). Indeed, the main differences between the two FoPLs are that NutrInform is per portion (vs. Nutri-Score per 100 g) and that Nutri-Score takes into account foods and nutrients to favor as well (vs. only nutrients to limit for NutrInform). To assess **purchase intention**, we followed the same methodology as for objective understanding to rank products according to their nutritional qualities. We attributed 5 points to the most nutritionally favorable product according to the FoPL (e.g., 5 points attributed to oat flakes in the Nutri-Score group and 5 point for wholegrain biscuit in the NutrInform group) and 1 point to the product with the lowest nutritional quality (1 up to 3 points in the case of added fats due to lower nutritional variability). Some products could have the same score and there were equal chances between score options of both FoPLs groups.

Multivariable ordinal and binary logistic models were used to assess the associations between the ability to choose the three correct products (*three-product task*) or select the expected product in the first position (*one-product task*) respectively with Nutri-Score compared to NutrInform (reference). Socio-demographic and context variables displaying statistical significance at the *p*-value < 0.20 level in bivariate models were included in the multivariable model [25]. Models were adjusted for sex, educational level, presence of children in the household, and understanding of the information note grade. Statistical analyses were carried out using the full sample of participants for all food categories combined and by individual food category.

The associations between the intention to buy higher nutritional quality products and randomization group (NutrInform as a reference) were evaluated through multivariable ordinal logistic models adjusted for sex, educational level, presence of children in the household, and understanding of the information note grade. Variables displaying statistical significance at the *p*-value < 0.20 level in bivariate models were included in the multivariable model [25]. Statistical analyses were carried out on full sample except participants who selected the “None of these products” option available for the breakfast products and the breakfast cereals categories.

To analyze the last two questions exposing all participants to Nutri-Score and NutrInform (**direct preference**), we used the variable measuring whether participants preferred the FoPL to which they were assigned over the newly presented FoPL (e.g., they responded Nutri-Score while in the Nutri-Score randomization group). Multivariable logistic regression models were fitted to assess the associations between “preferred the FoPL they were assigned to” and the randomization group, using the NutrInform group as reference. The models were adjusted for sex, educational level, presence of children in the household and having heard negative things about Nutri-Score.

All statistical tests were bilateral and a *p*-value below 0.05 was considered significant. All tests were conducted using R Software (version 3.4.4, R Foundation, Vienna, Austria).

## 3. Results

In the sample of 1064 Italian respondents, mean age was 46.5 ± 14.1 years old, 48% were men, and 38% had a university degree. Overall, 23% declared having an unhealthy diet and 26% reported having poor knowledge of nutrition. Regarding the FoPL familiarity, more participants declared having heard about NutrInform before (54% vs. 43% for Nutri-Score), and mainly in a positive way for both FoPLs (60% for Nutri-Score and 73% for NutrInform, among people who had heard about FoPL before). Regarding the understanding of the information note, it appeared that participants better understood how Nutri-Score worked compared to NutrInform (4.38 ± 2.19, 3.03 ± 1.39, respectively; *p* < 0.0001). Data per randomization group are detailed in Table 1.

In terms of **subjective understanding** (Table 2), Nutri-Score was perceived to be more helpful for distinguishing the nutritional quality of breakfast cereals than NutrInform (1.15 ± 1.05 vs. 1.02 ± 0.91, *p* = 0.04). No significant differences were observed between the two randomization groups for the remaining food categories. In terms of overall **perception**, Nutri-Score was perceived to be easier to use than NutrInform (0.22 ± 1.63 vs. −0.23 ± 1.66, *p*<0.0001), notably because it was easier to understand/interpret and less confusing. Other perception variables were not significantly different between the FoPLs.

As to the **objective understanding** of the two FoPLs, Nutri-Score was associated with a higher ability of participants to identify the correct products in all product categories (Table 3). The strongest odds ratios were observed for the following tasks: selecting the most nutritionally favorable added fat (OR = 21.7 [15.3–31.1], *p* < 0.0001) and identifying the three correct breakfast products (OR = 12.9 [9.64–17.2], *p* < 0.0001). For the *one-product task*, the overall performance across all food categories was at OR = 14.1 [10.6–18.6], *p* < 0.0001 in favor of the Nutri-Score.

Regarding **purchase intention**, being in the Nutri-Score group was associated with higher odds of buying products with more favorable nutritional quality compared to the NutrInform group (across all food categories, OR = 5.29 [4.02–6.97], *p* < 0.0001). Moreover, additional analysis (Table S3) showed that products selected by the Nutri-Score group participants were significantly lower in sugars, salt and saturated fats. For the specific case of olive oil, 83% of participants in the Nutri-Score group declared they would buy olive oil more frequently vs. 66% in the NutrInform group (data not tabulated, OR = 1.92 [1.42–2.60], *p* < 0.0001).

Finally, in the last part of the questionnaire, among all participants, 70% preferred Nutri-Score. In the Nutri-Score group, 30% responded in favor of NutrInform, whereas in the NutrInform group, 46% responded in favor of Nutri-Score (data not tabulated). Of note, compared to the NutrInform group, participants in the Nutri-Score group who declared having heard negative things about Nutri-Score (*n* = 72) had lower odds of responding in favor of Nutri-Score (OR = 0.21 [0.10–0.42], *p* < 0.001).

Being in the Nutri-Score group significantly increased the odds of preferring the FoPL to which the participant was primarily exposed (OR = 1.81 [1.41–2.34]; OR = 2.13 [1.66–2.75], *p* < 0.0001 for direct preference 1 and 2, respectively) (Table 4).

## 4. Discussion

This study showed a higher performance for Nutri-Score, compared to NutrInform, in helping participants identify the most nutritionally favorable products in an experimental online choice task, leading to stronger intentions to purchase products with higher nutritional qualities in the future.

High performances of Nutri-Score in objective understanding tasks (food choices, ranking) against non-interpretive labels such as Reference Intakes (graphic format close to NutrInform) have been demonstrated in the past, including in Italy [26,27]. However, to date, no other study has directly compared performances of Nutri-Score and NutrInform on objective understanding and purchase intention. One study by Mazzù et al. [20] focused only on the perception of these two labels tested via a questionnaire on 200 Italian participants, in a real-life setting. They concluded that NutrInform was perceived as an informative FoPL by consumers in terms of understanding of the product composition and that it performed better than Nutri-Score on all the perception dimensions studied (Comprehensibility, Help-to-shop, Complexity, and Liking). In our study, perception scores between the two FoPLs were close; nevertheless, Nutri-Score was perceived as being easier to use compared to NutrInform. In Mazzù et al., this dimension was entitled “Help to shop”, and notably included the item “this label makes it easier to choose food” for which, contrary to our results, NutrInform obtained a better score. This might be explained by the choices of authors in grouping and formulating perception items and/or the manipulation of the FoPL prior to perception tasks. Inconsistency in perception results across studies calls for the necessity of manipulation tasks to analyze the impact FoPLs can have on consumers food choices. However, comparing performance of non-interpretive, nutrient-specific FoPLs with interpretive summary indicators can be challenging as FoPLs such as NutrInform do not rank food products according to their overall nutritional quality and that products “to favor” can depend on the objectives of the consumers [20]. Nevertheless, dietary intakes of nutrients to limit (added sugar, sodium, and SFA) are considered a high public health priority in a majority of European countries [1] and FoPLs should encourage food choices going towards a reduction of these nutrients. The results of our study suggest that Nutri-Score is more effective in directing consumers to products of lower contents in nutrients of concern than NutrInform.

Looking at the links between subjective and objective understanding, Nutri-Score was perceived as being more helpful than NutrInform in discriminating between the nutritional quality of products in the breakfast cereals category only (1.15 ± 1.05 vs. 1.02 ± 0.91, *p* = 0.04). This finding did not particularly influence performance, as higher ORs were observed for the added fats category (OR = 21.7 [15.3–31.1] and OR = 33.2 [23.3–47.5], *p* < 0.0001 for objective understanding and purchase intention, respectively). Finally, when participants were confronted with both FoPLs affixed on three types of cookies, overall, 70% of participants found Nutri-Score easier and faster to make choices based on nutritional quality. These additional findings highlight the difference in effect size between consumer preference and performance results. Furthermore, they support the need for performance measures, as preference measures do not accurately reflect performance and do not prejudge the actual effects of FoPLs in manipulation tasks. In its manual for developing FoPLs, the WHO states that: “The key study to conduct is the investigation of consumers’ objective understanding [relatively to subjective understanding]” [17] in order to compare graphical formats of FoPLs.

One of the main criticisms of Italian stakeholders in the debate on the Nutri-Score is that it would wrongly penalize products from the Mediterranean diet, particularly olive oil [15]. Therefore, we included in this study the food category added fats to analyze the impact of Nutri-Score and NutrInform on objective understanding and purchase intention of participants. It appears that the two FoPLs classify added fats differently. In the case of NutrInform, the visual parameters allowing the discrimination between these products are fats and saturated fats battery levels only. Of the seven added fats selected for the study, rapeseed and sunflower oil had lower saturated fat contents compared to olive oil (0.8 g and 1 g for a portion of 10 g compared to 1.6 g per 10 g for olive oil, respectively). In the case of Nutri-Score, the best options were olive or rapeseed oils, both rated C (the best grade for added fats). NutrInform’s graphic format resulted in lower olive oil purchase intention among participants compared to Nutri-Score (66% vs. 83%, respectively, declared they would buy olive oil the most frequently). In a study among Spanish consumers [28], we found that Nutri-Score did not negatively impact the image of olive oil among participants, as a majority stated they would still consume it and thought Nutri-Score should be displayed on olive oil. In the end, Nutri-Score on added fats seems to be well accepted by participants, and it appears to direct consumers’ food choices towards olive oil in a stronger way than NutrInform.

Regarding trends associated with the Mediterranean diet, in 2015, the Food and Agriculture Organization of the United Nations (FAO) released a report analyzing Mediterranean food consumption patterns [29]. One of the findings was the decline in Mediterranean diet adherence, particularly in the youth: “The Mediterranean diet pattern is presently in decline among consumers because of standardization of lifestyles, loss of awareness and appreciation, particularly among younger generations, about their own cultural food heritage” [29]. In Italy, the same trends have been noticed with a shift towards more sugary and refined foods. Denoth et al. [11] found that, of the 5278 participants, fewer than half were eating a Mediterranean-style diet (mainly women and the elderly), while the rest followed either a “Western-like” diet or a diet that was low in fruits and vegetables. Our study showed that Nutri-Score was more efficient compared to NutrInform in orienting participants towards products with better nutritional quality that tend to be less sugary (e.g., oats or crispbread instead of packaged chocolate croissants for the breakfast products category; plain oats instead of refined sugary cereals in the breakfast cereals category). Additionally, NutrInform only highlights the content of nutrients to be limited, while Nutri-Score’s algorithm includes also nutrients and foods that are to be favored, which are promoted in the Mediterranean diet [13,30] and for which intakes tend to be too low in the European population [1]. With an appealing and accessible format [31,32], Nutri-Score could be an interesting tool for younger individuals in order to deter them from the consumption of products high in sugar, salt and saturated fats.

Strengths of our study include the investigations of various dimensions of FoPL analysis such as objective understanding (*one-product* and *three-product* tasks), purchase intention and subjective understanding on three food categories, and overall perception. The form of the online questionnaire allowed us to include participants of different levels of education and a wide range of ages, although those who chose to complete the questionnaire may have been more interested in food/nutrition-related topics. Participants were presented with an information note on the FoPL they were assigned to (accessible throughout the questionnaire), covering a wide range of the FoPL characteristics and allowing the respondent to get familiar with the FoPL before replying to performance and preference sections. The commitment to consider each FoPL’s special features in defining the correct answers in objective understanding tasks allowed us to ensure equitable chances across the two randomization groups. 

Limitations of our study include the fact that the debate on Nutri-Score in Italy, with mediatized campaigns by the agri-food sectors positioning Nutri-Score as a threat to Italian traditional foods, started prior to this investigation, potentially affecting some respondents’ answers. Nevertheless, this potential bias was considered by adjusting the results of direct preference (Table 4) with participants who declared having heard negative things about Nutri-Score (*n* = 72), as they had higher odds of responding in favor of NutrInform. We decided to use images of real-life products instead of mock packages to increase the questionnaire’s plausibility. However, this may have affected answers of participants according to their familiarity with the food products. In addition, our study was limited to 23 products of three specific food categories, representing only part of the diet. 

## 5. Conclusions

In conclusion, this study brings new results in the comparison between the interpretive, summary label Nutri-Score and the non-interpretive, nutrient-specific FoPL NutrInform, assessing both performance and consumer preference dimensions, thus complementing existing studies focusing on consumer preference only. In the current context of the pending proposal for a harmonized mandatory label at EU level and a strong debate in Italy on FoPLs involving both economic and political stakeholders, this study provides new evidence as to whether Nutri-Score or NutrInform would be better able to “support consumers to choose nutritionally favorable products” as stated by WHO. Indeed, we showed that Nutri-Score, perceived as easier to use than NutrInform among Italian participants, led to higher intentions to purchase nutritionally favorable products. Future studies could compare the two FoPLs’ performance on other food categories and in real-life settings.

## Figures and Tables

**Table 1:** Individual characteristics of participants, context and understanding of the information note per randomization group (*n* = 1064).

	Nutri-Score Group (*n* = 532)		NutrInform Group (*n* = 532)	
	N	%	N	%
**Sex**				
Men	256	48%	256	48%
Women	276	52%	276	52%
**Age categories, years**				
18–34	124	23%	127	24%
35–54	229	43%	226	42%
55–80	179	34%	179	34%
**Educational level**				
No university degree	326	61%	329	62%
University degree	206	39%	203	38%
**Presence of children (≤13 yo) in the household**				
Without children	371	70%	359	67%
With children	161	30%	173	33%
**Self-estimated diet quality**				
Unhealthy diet	131	25%	109	20%
Healthy diet	401	75%	423	80%
**Self-estimated nutrition knowledge**				
Poor knowledge of nutrition	136	26%	141	27%
Good knowledge of nutrition	396	74%	391	73%
**Did you hear about Nutri-Score before?**				
No	311	58%	299	56%
**If yes, what you heard was…**				
Neutral	56	11%	52	10%
Negative	37	7%	35	7%
Positive	128	24%	146	27%
**Did you hear about NutrInform before?**				
No	263	49%	228	43%
**If yes, what you heard was…**				
Neutral	64	12%	70	13%
Negative	9	2%	10	2%
Positive	196	37%	224	42%
**Understanding of the information note** ^**1**^	Mean grade = 4.38 ± 2.19		Mean grade = 3.03 ± 1.39	

Headings in bold define the categories of questions in the questionnaire. ^1^ refers to the consumer’s ability to correctly answer seven questions about the information note (grade out of seven).

**Table 2:** Results of subjective understanding by food category and overall perception (*n*=1064).

	Nutri-Score (*n* = 532)	NutrInform (*n* = 532)	
	Mean ± SD	Mean ± SD	*p*-Value
Subjective understanding ^1^			
Breakfast products	1.20 ± 0.98	1.19 ± 0.87	0.9
Breakfast cereals	1.15 ± 1.05	1.02 ± 0.91	**0.04**
Added fats	0.89 ± 1.16	0.92 ± 1.02	0.7
Perception ^2^			
Ease of use	0.22 ± 1.63	−0.23 ± 1.66	**<0.0001**
Capacity to inform	−0.044 ± 1.74	0.046 ± 1.57	0.38
Trust	−0.045 ± 1.70	0.047 ± 1.53	0.36
Liking	−0.024 ± 1.25	0.025 ± 1.10	0.50

SD: standard deviation; boldface indicates statistical significance (*p* < 0.05). ^1^ refers to the reported helpfulness of the FoPL in discriminating the nutritional quality of products in each food category (the Likert scale was converted in a score from −2, Strongly Disagree to +2, Strongly Agree). ^2^ refers to mean coordinates of participants on the first PCA dimension of each perception group, standardized variable. The exclusion of participants responding “neither agree nor disagree” for all statements of a perception group led to the following total samples (in the same order as the table): *n* = 1043; *n* = 1040; *n* = 1020; *n* = 1004.

**Table 3:** Associations between Nutri-Score and the capacity to identify the most nutritionally favorable product; the intention to purchase products with a more favorable nutritional quality; the intention to purchase olive oil (*n* = 1064).

	Objective Understanding				Purchase Intention	
	One-Product Task		Three-Product Task			
	OR [CI]	*p*-Value	OR [CI]	*p*-Value	OR [CI]	*p*-Value
Breakfast products ^1^	6.13 [4.62–8.18]	<0.0001	12.9 [9.64–17.2]	<0.0001	1.81 [1.41–2.33]	<0.0001
Breakfast cereals ^1^	7.06 [5.29–9.50]	<0.0001	3.84 [2.95–5.00]	<0.0001	2.23 [1.70–2.92]	<0.0001
Added fats	21.7 [15.3–31.1]	<0.0001	-	-	33.2 [23.3–47.5]	<0.0001
All food categories	14.1 [10.6–18.6]	<0.0001	-	-	5.29 [4.02–6.97]	<0.0001
Olive oil	-	-	-	-	1.92 [1.42–2.60]	<0.0001

The multivariate logistical regression models (ref. NutrInform) were adjusted for sex, education level, presence of children in the household, understanding of the information note grade. OR: odds ratio; CI: 95% confidence interval; boldface indicates statistical significance (*p* < 0.05); “-”: for added fats, participants had to select only one product (due to a more limited difference in nutrient composition in this particular category), as a result the overall performance for the *three-product task* could not be assessed. ^1^ for the breakfast products and the breakfast cereals categories, the purchase intention section included an answering option “None of these products”. Removing these cases from the analysis reduced the sample to *n* = 939 for breakfast products and *n* = 880 for breakfast cereals.

**Table 4:** Association between Nutri-Score and the probability of preferring the FoPL the participant was mainly exposed to (*n* = 1064).

	OR [CI]	*p*-Value
Direct preference 1 ^1^	1.81 [1.41–2.34]	<0.0001
Direct preference 2 ^2^	2.13 [1.66–2.75]	<0.0001

The multivariate logistical regression models (ref. NutrInform) were adjusted for sex, education level, presence of children in the household, heard negative things about Nutri-Score. OR: odds ratio; CI: 95% confidence interval; boldface indicates statistical significance (*p* < 0.05). ^1^ Between the Nutri-Score and NutrInform nutrition information labels, which one makes it easier for you to assess the differences in nutritional quality between these products? ^2^ Which label would you like to see on food packaging to help you quickly find the product with better nutritional quality?

## Data Availability

The datasets used and/or analyzed during the current study are available from the corresponding author on reasonable request.

## Supplementary Materials

The following supporting information can be downloaded at: https://www.mdpi.com/article/10.3390/nu14173511/s1, Figure S1: Objective understanding task for the breakfast products category; Table S1: List of correct answers for objective understanding tasks; Table S2: Summary of the information notes on Nutri-Score and NutrInform Battery provided to participants at the beginning of the questionnaire; Table S3: Average contents of nutrients of concern (g/100 g) per FoPL group and food category based on purchase intentions of participants.
