# Supplementary material for: Nutri-Score and NutrInform Battery: Effects on Performance and Preference in Italian Consumers
Source: Nutrients. 2022 Aug 26;14(17):3511. doi: 10.3390/nu14173511 (PMC9459720; doi:10.3390/nu14173511)
Supplement: Supplementary file 1 [file nutrients-14-03511-s001.zip › nutrients-1850633-supplementary.pdf]

Figure S1: Objective understanding task for the breakfast products category

Con l'aiuto del Nutri-Score, quali alimenti considera di <u>più alta qualità nutrizionale</u>?

Posizionandosi sui prodotti o sul logo, appare una lente d'ingrandimento che aiuta a visualizzare meglio le immagini

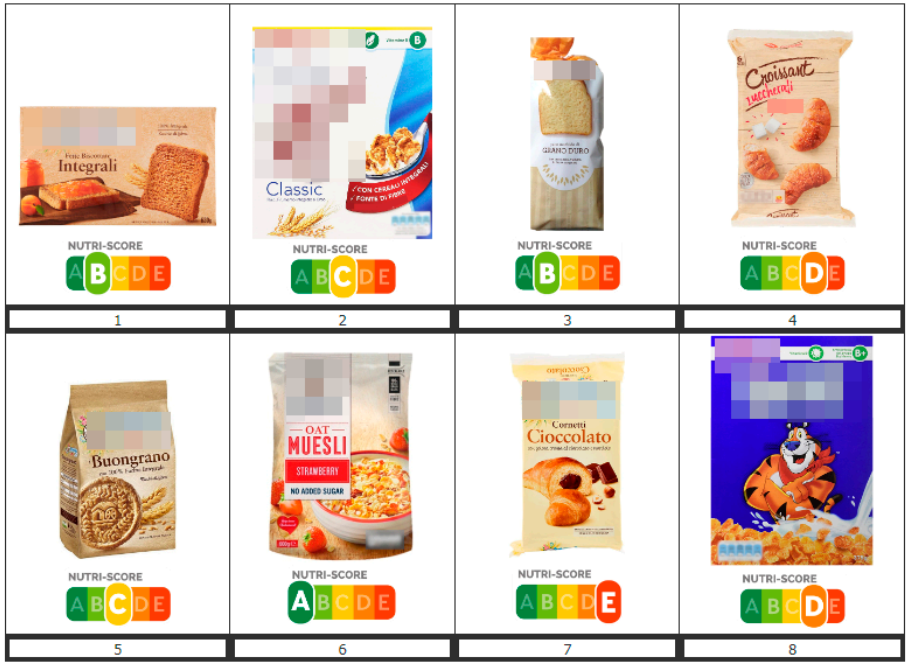

selezionare tre numeri tra quelli posizionati sotto il prodotto corrispondenti ai 3 prodotti che si ritiene abbiano le migliori qualità nutrizionali, **mettendo nella prima casella** quello con la migliore qualità nutrizionale

I 3 prodotti con le migliori qualità nutrizionali:			

Quale prodotto comprerebbe più spesso?	
Nessuno	

Nota informativa

Con l'aiuto del NutrInform, quali alimenti considera di <u>più alta qualità nutrizionale</u>?

Posizionandosi sui prodotti o sul logo, appare una lente d'ingrandimento che aiuta a visualizzare meglio le immagini

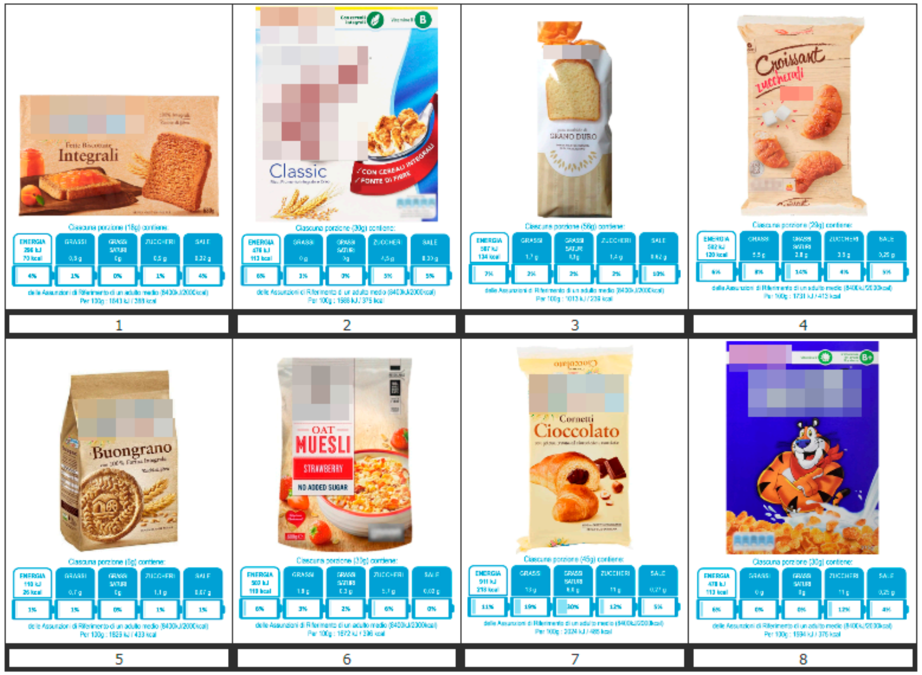

selezionare tre numeri tra quelli posizionati sotto il prodotto corrispondenti ai 3 prodotti che si ritiene abbiano le migliori qualità nutrizionali, **mettendo nella prima casella** quello con la migliore qualità nutrizionale

I 3 prodotti con le migliori qualità nutrizionali:			

Quale comprerebbe più spesso?	
Nessuno	

Nota informativa

Table S1: Summary of the information notes on Nutri-Score and NutrInform Battery provided to participants at the beginning of the questionnaire

Content	Nutri-Score	NutrInform Battery
**Description**	A nutritional label intended to be placed on the front of food packages to inform consumers of the overall nutritional quality of foods	A nutritional label intended to be placed on the front of food packages to inform consumers about the nutritional composition of foodstuffs
**Developers**	Independent academics and researchers specialized in nutrition	The Italian Ministries of Health, Agriculture, Food and Forestry Policies and Economic Development, with the support of government agencies
**Implementation**	Adopted by public health authorities in several countries, among them France, Belgium, Germany	Adopted by the Italian public health authorities
**Calculation**	Its calculation takes into account the balance between the unfavorable elements contained in 100g of each solid food or 100 ml of beverage (sugars, saturated fats, salt, calories) and the favorable elements (fiber, proteins, presence of fruits, vegetables, legumes, nuts, rapeseed, walnut and olive oil).	NutrInform is a nutritional information system that informs you, on the basis of a portion of the product, of the quantity and percentage of calories, sugars, lipids, saturated fats and added salt in relation to the daily recommendation for an adult (2000Kcal).
**Format**	The Nutri-Score is presented in the form of a 5-color label ranging from green/A (highest nutritional quality) to red/E (lowest nutritional quality).	The NutrInform label graphically represents the percentage of calories and various nutrients in relation to the recommended serving size of the food. The battery level is the percentage of each nutrient provided by the portion of foodstuff compared to the average daily recommendation for an adult.
**How to use it**	Nutri-Score only makes it possible to compare the nutritional quality of foods that can be consumed under the same conditions (for example, between foods eaten for breakfast or as a snack or dessert, or those eaten as a main course...) and thus to be able to assess at a glance whether a food has a better or worse nutritional quality than another food that is comparable in use.	It is necessary to take care not to "overload" the batteries corresponding to the various nutrients and energy, considering the other foods, and therefore the nutrients and calories consumed in a day.

Table S2: List of products in each food category with their corresponding Nutri-Score and NutrInform and the expected answers for objective understanding

Answers [1]			Nutri-Score	NutrInform					
Nutri-Score	NutrInform	Product	Grade	Portion	Energy	Fats	Saturated fats	Sugars	Salt
		Breakfast products							
1	2	Muesli flakes with fruits	A	30g	6%	3%	2%	6%	0%
2	2	Crispbread	B	18g	4%	1%	0%	1%	4%
2		Sliced bread	B	56g	7%	2%	2%	2%	10%
	1	Wholegrain biscuits	C	6g	1%	1%	0%	1%	1%
		Diet cereals	C	30g	6%	1%	0%	5%	5%
		Plain croissant	D	29g	6%	8%	14%	4%	5%
		Child-targeted sweet cereals	D	30g	6%	0%	0%	12%	4%
		Chocolate croissant	E	45g	11%	19%	30%	12%	5%
		Breakfast cereals							
1	1	Oat flakes	A	30g	6%	3%	2%	0%	1%
2	2	Corn flakes	B	30g	6%	0%	0%	3%	6%
2		Child-targeted sweet cereals	B	30g	6%	2%	2%	8%	1%
	2	*Diet* cereals	C	30g	6%	1%	1%	4%	5%
		Child-targeted sweet cereals	D	30g	6%	2%	1%	9%	5%
		Child-targeted sweet cereals	D	30g	6%	1%	3%	13%	7%
		Chocolate cereals	E	30g	7%	9%	15%	8%	3%
		Added Fats							
1	1	Rapeseed oil	C	10g	4%	14%	4%	0%	0%
1		Olive oil	C	10g	4%	14%	8%	0%	0%
	1	Sunflower oil [2]	D	10g	4%	14%	5%	0%	0%
		Soybean oil	D	10g	4%	14%	8%	0%	0%
		Peanut oil	D	10g	4%	14%	9%	0%	0%
		Corn oil	D	10g	4%	14%	7%	0%	0%
		Butter	E	10g	4%	12%	29%	0%	0%
		Palm oil	E	10g	4%	14%	25%	0%	0%

[1] The number 1 corresponds to the answer for one-product task and number 2 corresponds to the answers for three-product task (in addition to number 1)

[2] Although rapeseed oil could be considered as the only correct answer in the case of NutrInform (4% saturated fats), sunflower oil (5% saturated fats) was also considered as correct in order to ensure equivalent chances of obtaining a correct answer in both groups.

Table S3: Average contents of nutrients of concern (g/100 g) per FoPL group and food category based on purchase intentions of participants

	x/100g	**Nutri-Score**	**NutrInform**	**p-value**[2]
**Breakfast products** [1] (n=939)	*SFA (g)*	1,61	2,14	**0,02**
	Sugars (g)	13,20	14,93	**0,001**
	Salt (g)	0,69	0,95	**<0,0001**
**Breakfast cereals** [1] (n=880)	*SFA (g)*	1,44	2,12	**0,0003**
	Sugars (g)	11,27	13,77	**0,0004**
	Salt (g)	0,54	0,76	**<0,0001**
**Added fats** (n=1064)	*SFA (g)*	16,43	17,87	**0,01**

[1] for the breakfast products and the breakfast cereals categories a "None of these products" option was available for the purchase intention section

[2] results of t-test (Standard/Welch according to variance); boldface indicates statistical significance (p<0.05)
